# Autocleavage of the paracaspase MALT1 at Arg-781 attenuates NF-κB signaling and regulates the growth of activated B-cell like diffuse large B-cell lymphoma cells

**DOI:** 10.1371/journal.pone.0199779

**Published:** 2018-06-28

**Authors:** Chun-Hsien Wu, Yu-Hsuan Yang, Mei-Ru Chen, Ching-Hwa Tsai, Ann-Lii Cheng, Shin-Lian Doong

**Affiliations:** 1 Graduate Institute of Microbiology, College of Medicine, National Taiwan University, Taipei, Taiwan; 2 Department of Oncology and Internal Medicine, National Taiwan University Hospital, Taipei, Taiwan; 3 Cancer Research Center, College of Medicine, National Taiwan University, Taipei, Taiwan; J. Heyrovsky Institute of Physical Chemistry, CZECH REPUBLIC

## Abstract

MALT1 controls several receptors-mediated signaling to nuclear factor κB (NF-κB) through both its scaffold and protease function. MALT1 protease activity is shown to inactivate several negative regulators of NF-κB signaling and augment NF-κB activation ability. In this study, MALT1 was demonstrated to autoprocess itself in the presence of oligomerization-competent BCL10. Cleavage occurred after Arginine 781 located in the C-terminus of MALT1. Shortened MALT1 cleavage products showed attenuated binding ability with TRAF6. Its NF-κB activation ability was also weakened. Various MALT1 constructs including wild type, catalytically-inactive (MALT1_C464A), cleavage-defective (MALT1_R781L), or truncated (MALT1_1–781) form of MALT1 was introduced into MALT1-knocked-down-Jurkat T cells. Cleavage-defective MALT1_R781L retained its proteolytic and initial IκBα phosphorylation activity as MALT1. Truncated MALT1_1–781 mutant showed weakness in IκBα phosphorylation and the expression of NF-κB targets IL-2 and IFN-γ. Cleavage at R781 was detectable but marginal after activation with TPA/ionomycin or anti-CD3 antibody in lymphocytes. However, cleavage at R781 was evident in ABC-DLBCL cells such as OCI-Ly3, HBL-1. HBL-1 cells with induced expression of catalytically-inactive MALT1_C464A or cleavage-defective MALT1_R781L exhibited characteristic of retarded-growth. These findings suggested that cleavage at R781 of MALT1 played a role in the survival of ABC-DLBCL cells.

## Introduction

Human MALT1 (Mucosa-associated lymphoma translocation 1) contains 824 amino acid residues with an N-terminal death domain, two Ig (immunoglobulin)-like domains, followed by a CLD (caspase-like-domain) and a third Ig-like domain [1,2]. Upon receptor stimulation, the relevant CARMA (CARD containing membrane associated protein) recruits BCL10 and MALT1, known as CBM complex, to trigger NF-kB activation [3]. The CBM complex is thought to oligomerize MALT1 [4] and its associateddownstream factor TRAF6, which in turn facilitates k63-linked poly-ubiquitination of several proteins including TRAF6 [5], BCL10 [6] and MALT1 [7]. Poly-ubiquitination of these proteins leads to the recruitment of TAk1 (transforming growth factor β-activated kinase 1), TAk1 binding protein (TAB), and the Ikk complex to lipid rafts where the Ikk β-subunit is phosphorylated and activated. The activated Ikk complex phosphorylates IkB, enabling proteasome-mediated degradation of IkB and subsequent translocation of NF-kB into the nucleus and induces the downstream gene expression.

Besides its first-identified scaffolding function, MALT1 has arginine-specific proteolytic activity [8,9]. The catalytic activity of MALT1 and the biological consequences resulting from its proteolytic activation have been topics of great interest. Numerous MALT1 substrates have been identified [1]. BCL10 was the first identified proteolytic substrate of MALT1 [10]. However, proteolytic processing of BCL10 is associated with the fibronectin adhesion and not required for NF-kB activation [10]. Many among those identified substrates are negative regulators in NF-kB signaling, like A20 [11], RelB[12], Regnase-1 [13] and Roquins[14]. MALT1 was reported to be its own substrate [15]. The auto-cleavage at R149 of MALT1 is important for NF-kB downstream target genes expression in T and B cells [15]. Collectively, MALT1-mediated cleavage of these substrates are believed to enhance and prolong NF-kB signaling. Lately, HOIL-1 was identified as MALT1 substrate [16–18]. In contrast to other MALT1 substrates, the cleavage of HOIL-1 was demonstrated to be involved in the negative feedback regulation of LUBAC-dependent NF-κB signaling [16,18].

The ABC (activated B cell) subtypes of *diffuse large B-cell lymphoma* (DLBCL) are characterized by constitutive NF-kB signaling [19]. The activated NF-kB signaling pathway is known to be essential for the survival of ABC-DLBCL [20]. Since CARMA1/BCL10/MALT1 signaling pathway was reported to play key roles in the activation of NF-kB in these ABC-DLBCL cells. Inhibition of the protease activity of MALT1 was found to be able to inhibit the growth of ABC-DLBCL cells [21–24]. These studies successfully demonstrated the essential role of the proteolytic activity of MALT1 in NF-kB activation and proliferation of ABC-DLBCL cells.

We have been interested in studying mechanisms involved in the regulation of MALT1. In 293T cells, over expression of BCL10 with MALT1 triggers the proteolytic activity of MALT1. In addition to the cleavage of BCL10, we consistently observed the appearance of a faster migrating MALT1 fragment. A cleavage site at R781 of MALT1 was identified. While the manuscript was in preparation, Ginster *et al*. [25] also reported the identification of R781 as the auto-proteolysis site of MALT1, focusing on mechanisms mediated by TRAF6 on MALT1 self cleavage reaction and function.Yet, the biological significance of self cleavage at R781 of MALT1 awaits for determination. In this study, we revealed the role of the cleavage in the growth of ABC-DLBCL cells.

## Materials and methods

### Plasmids and antibodies

Expression vectors pCMV6/XL5/MALT1 [containing full length isoform A (824 aa) MALT1 cDNA] was purchased from ORIGENE Technologies Inc. (Rockville, Maryland). pcDNA3.1^**+**^/C-(k)DYk-MALT1B [containing full length isoform B (813 aa) MALT1 cDNA] was purchased from GenScript (Piscataway, NJ). Schematic representation of all MALT1 variants was shown in S1 Fig. Please also see the S1 Text for detailed construction information.

The following antibodies were used: anti-MALT1 (Santa Cruz Biotechnology, B-12 sc-46677, mouse monoclonal), anti-MALT1 (Santa Cruz Biotechnology, H-300 sc-28246, rabbit polyclonal), anti-BCL10 (Santa Cruz Biotechnology, 331.3 sc-5273, mouse monoclonal), anti-IκBα (Santa Cruz Biotechnology,C-15 sc-203, rabbit polyclonal), anti-phospho-IκBα (Cell Signaling Technology #9246, mouse monoclonal), anti-RelB (Cell Signaling Technology #4922, rabbit monoclonal), anti-Regnase-1 (MCPIP1) (R&D Systems #604421, mouse monoclonal), anti-HA (Santa Cruz Biotechnology, F-7 sc-7392, mouse monoclonal), anti-FLAG (Sigma M2, mouse monoclonal), anti-GAPDH (Santa Cruz Biotechnology, 6C5 sc-32233, mouse monoclonal), goat antiserum to human IgG (MP Biomedicals, LLC. #55087), peroxidase-labeled goat anti-mouse IgG (SeraCare Life Science, kPL 074–1806), peroxidase-conjugated goat anti-rabbit IgG (Jackson ImmunoResearch Inc. 111-035-003).

### Cell culture

HEk293T cells (ATCC^®^ CRL-3216^TM^) were cultured in DMEM containing 10% (vol/vol) FBS, 50 units/ml penicillin, 50 μg/ml streptomycin, 1.25 μg/ml fungizone (all from GIBCO, Invitrogen, Carlsbad, CA). Jurkat (kindly provided by Dr. Y.C. Yang at the Department of Clinical Laboratory Sciences and Medical Biotechnology, College of Medicine, National Taiwan University), BJAB and EBV^**+**^-Akata (gift from Dr. kenzo Takada) cells were cultured in RPMI 1640 with the same supplements. Lentivirally-transduced Jurkat cells were kept in the presence of 2 μg/ml puromycin or/and 1 mg/ml G418. The human DLBCL cell lines (kindly provided by Dr. J.T. Hsieh, University of Texas Southwestern Medical Center), SUDHL-4, SUDHL-6, HBL-1, OCI-Ly3, and U2932, were cultured in IMDM supplemented with 10% FBS and 50 units/ml penicillin, 50 μg/ml streptomycin, 1.25 μg/ml fungizone. All cells were cultured at 37°C in a 5% CO_2_ incubator. Splenocytes of wild type C57BL/6 mice and MALT1 knock-out C57BL/6 mice [26] were kindly provided by Dr. B.A.Wu-Hsieh at the Graduate Institute of Immunology, College of Medicine, National Taiwan University.

### Transfection in HEk293T

In a 6-well tissue culture plate, 2 × 10^5^cells/well were transfected by the calcium phosphate-DNA precipitation method. Sixteen hours later cells were washed with PBS and incubated at 37°C for another 24 hours before lysis.

### Western blot analysis

Cells were rinsed with PBS and lysed with RIPA buffer (50 mMTris-HCl, 1% NP-40, 0.1% SDS, 1mM EDTA, 1X protease inhibitor, 1mM Na_3_VO_4_, 1mM NaF). Denatured protein were separated on SDS/polyacrylamide gels (8%, 12% or 15%), and then transferred onto a polyvinylidenedilfluoride membrane (AmershamHybond P, GE Healthcare and Life Sciences) and incubated with indicated antibodies for 18 hr at 4°C, washed and then secondary antibodies for 1 hr at room temperature.

### Immunoprecipitation and CIAP treatment

Cells were rinsed with PBS and lysed with modified RIPA buffer (150 mM NaCl, 50 mMTris pH7.5, 1% NP40, 0.25% Sodium deoxycholate, 1mM EDTA, 1mM PMSF, 1mg/ml aprotinin, leupeptin, pepstatin, 1mM Na_3_VO_4_, 1mM NaF). 1 mg total cellular protein were incubated with 5 μl mouse anti-BCL10 antibody (sc-5273, Santa CrutzBiotchnology) for 16 hr at 4°C. 50 μl protein A beads were then added. The whole mixtures were incubated at 4°C for 4 hr. Protein A beads were spun down, washed twice with RIPA buffer and subjected to Calf Intestine Alkaline Phosphatase (CIAP) (New England Biolab.) treatment as suggested by the manufacturer’s protocol.

### Protein expression and purification

pET21aBCL10-His was transformed into BL21(DE3) *E*. *coli* cells. Protein expression was induced with 1 mM IPTG (isopropyl β-D-thiogalactopyranoside) for 4 hr at 37°C. *E*. *coli* cells were lysed in lysis buffer (50 mM NaH_2_PO_4_ pH 8.0, 300 mM NaCl, 10 mM imidazole, 8 M urea), sonicated. The lysates were centrifuged at 13k rpm (kUBOTA 1920) for 10 min at 4°C. The soluble fraction was applied to Ni^2+^ NTA agarose (Qiagen). Purification was performed as the manufacture’s instruction. The protein was eluted with 250 mM imidazole.

pET21a-MALT1-His or pET21a-MALT1_C464A-His was transformed into Arctic-Express^TM^ RIL compent *E*. *coli* cells. Protein expression was induced with 1 mM IPTG (isopropyl β-D-thiogalactopyranoside) for 48 hr at 8°C. *E*. *coli* cells were suspended in buffer (50 mM NaH_2_PO_4_ pH 8.0, 300 mM NaCl, 10 mM imidazole) and lysed by 700 psi french press (Thermo IEC FRENCH press laboratory with mini pressure cell, 120VAC, 60Hz). The lysates were centrifuged at 13k rpm (kUBOTA 1920) for 10 min at 4°C. The soluble fraction was applied to Ni^2+^ NTA agarose (Qiagen). The protein was purified according to the manufacture’s instruction and eluted with 250 mMimidazole.All the purified proteins were dialyzed against PBS and stored at –70°C freezer in the presence of 20% glycerol.

### *In vitro* cleavage assay of purified MALT1

50 ng purified BCL10 proteins were incubated with 1 μg or 2 μg purified full length MALT1 or catalytic-inactive mutant MALT1_C464A respectively for 4 hr at 30°C in 50 mM MES (pH 6.8), 150 mMNaCl, 10% sucrose, 0.1% CHAPS, 10 mM DTT, and 1 M ammonium citrate. The reaction was subsequently analyzed by SDS-PAGE, followed by Western blotting with an anti-BCL10 antibody or with an anti-MALT1 antibody.

### NF-κB reporter luciferase assays

HEk293T or Jurkat cells were transfected with plasmid DNAs encoding for NF-κB promoter-driven firefly luciferase (pNF-κB-Luc), and a *Renilla* luciferase (pRL-Tk) as the control. Jurkat T cells were transfected by electroporation using NeonTM transfection system (Invitrogen). 2 × 10^6^ cells in a total volume of 100 μl of serum free medium with the indicated plasmid DNA were electroporated using the following parameters (pulse voltage: 1325V, pulse width: 10 ms, pulse number: 3). HEk293T cells were transfected by the calcium phosphate-DNA precipitation method. 36 hrafter tansfection, the cells were lysed in 30 μl passive lysis buffer and the bioluminescence of the samples was measured using the Dual-Luciferase Reporter Assay System (Promega) and a Berthold Microplate Luminometer (Titertek Berthold). The relative luciferase activities were calculated after normalization of firefly luciferase activities to the activities of *Renilla* luciferase.

### Cell activation

T cell stimulation was performed by resuspending Jurkat T cells at a density of 1×10^7^ cells per ml in RPMI medium. TPA (10 ng/ml) plus ionomycin (1 μM) or anti-CD3 antibody (10 ng/ml) were then added and incubated at 37°C for indicated times. In some experiment, cells were preincubated for 30 mins at 37°C with 50 mM z-VRPR-fmk or 10 μM MG132 before stimulation. Splenocyte and BJAB activation were perfomed by treating cells at a density of 1×10^7^ cells per ml in RPMI medium with TPA (10 ng/ml) plus ionomycin (1 μM). BCR stimulation by cross-linkage of surface IgG in EBV^**+**^-Akata cells was performed by resuspending cells at a density of 2×10^6^ cells per ml in RPMI medium with 1% goat anti human IgG. At the end of the time course, cells were washed with PBS and then lysed in RIPA lysis buffer (100 μl for each 1×10^7^ cells).

### Transduction of cells, MALT1 RNA interference and rescue

RNAi reagents were obtained from the National Core Facility for Manipulation of Gene Function by RNAi, miRNA, miRNA sponges, and CRISPR / Genomic Research Center, Academia Sinica, supported by the National Core Facility Program for Biotechnology Grants of MOST (MOST 104-2319-B-001-001-).

For collection of recombinant lentivirus particles, 1 ml of HEk293T cells at a density of 2 × 10^5^ cells per ml were seeded on a 3.5-cm dish and transfected with 1.25 μg of lentiviral vector, 1 μg of pCMV-ΔR8.91, and 0.125μg of pMD.G. Twenty-four hours after transfection, cells were cultured with fresh DMEM medium containing 10% FBS for an additional 48 hrs. The culture medium containing lentivirus particles was centrifuged at 1000 ×*g* for 5 min. The supernatants were passed through 0.2 mm filter, collected and stored at −80°C.

For lentivirus infection, 5× 10^5^ cells were infected with sh-MALT1 lentivirus and selected by puromycin (2 μg/ml, Sigma). For the MALT1 rescue experiment, the MALT1 shRNA-positive cells were transduced separately with lentivirus particles generated from HEk293T cells transfected with various pLAS3.Pneo vector as0020described in supplementary data and selected by G418 (1 mg/ml, Bioman). For cell viability assay, HBL-1 cells were transduced separately with lentivirus particles generated from HEk293T cells transfected with various pAS4.1w.Ppuro-aOn MALT1 vector as described in supplementary data (S1 Text) and selected by puromycin (2 μg/ml, Sigma).

### ELISA

Human Jurkat T cells were treated with TPA (10 ng/μl) plus ionomycin (1 μM) for 8 hours. The supernatant of treated cells were collected. The IL-2 concentration was measured by human IL-2 ELISA MAX Deluxe Sets (Biolegend) according to manufacturer’s instruction.

### Quantitative real-time PCR

Total RNA was isolated from cells using TriPure reagent (Roche). cDNA synthesis was performed using the ToolsQuantII Fast RT kit (Tools) according to the manufacturer’s instructions. Quantitative PCR (qPCR) assays were performed with kAPA SYBR FAST qPCR Master Mix kit (kapa Biosystems) and analyzed using the comparative dCt method using GAPDH as a reference control. IL-2 mRNA was detected using forward primer 5’-TTCCTCCAGAGGTTTGAGTTCTT-3’ and reverse primer 5’-CTGCTGGATTTACACATGATTTT-3’. IFN-γ mRNA was detected using forward primer 5'-CTAATTATTCGGTAACTGACTTGA-3' and reverse primer 5'-ACAGTTCAGCCAATCACTTGGA-3'. GAPDH mRNA was detected using forward primer 5’-GAAGATGGTGATGGGA-3’ and reverse primer 5’-GAAGGTGAAGGTCGGAGTC-3’.

### Viability assays

HBL-1 cells were seeded in a 3.5-cm dish at a density of 2 × 10^5^ cells per ml and cultured in the absence or presence of 1 μg/ml doxycycline. Cells were set up in triplicate for each experiment. Three independent experiments were performed. Viable cells number was determined by trypan blue exclusion assay. Two-tailed Student’s *t*-test was applied for the experiment statistical analysis, and the confidence intervals were 95%. *P* values<0.05 was statically significant.

## Results

### BCL10 activates MALT1 and leads to self-cleavage of MALT1

**Fig 1:**
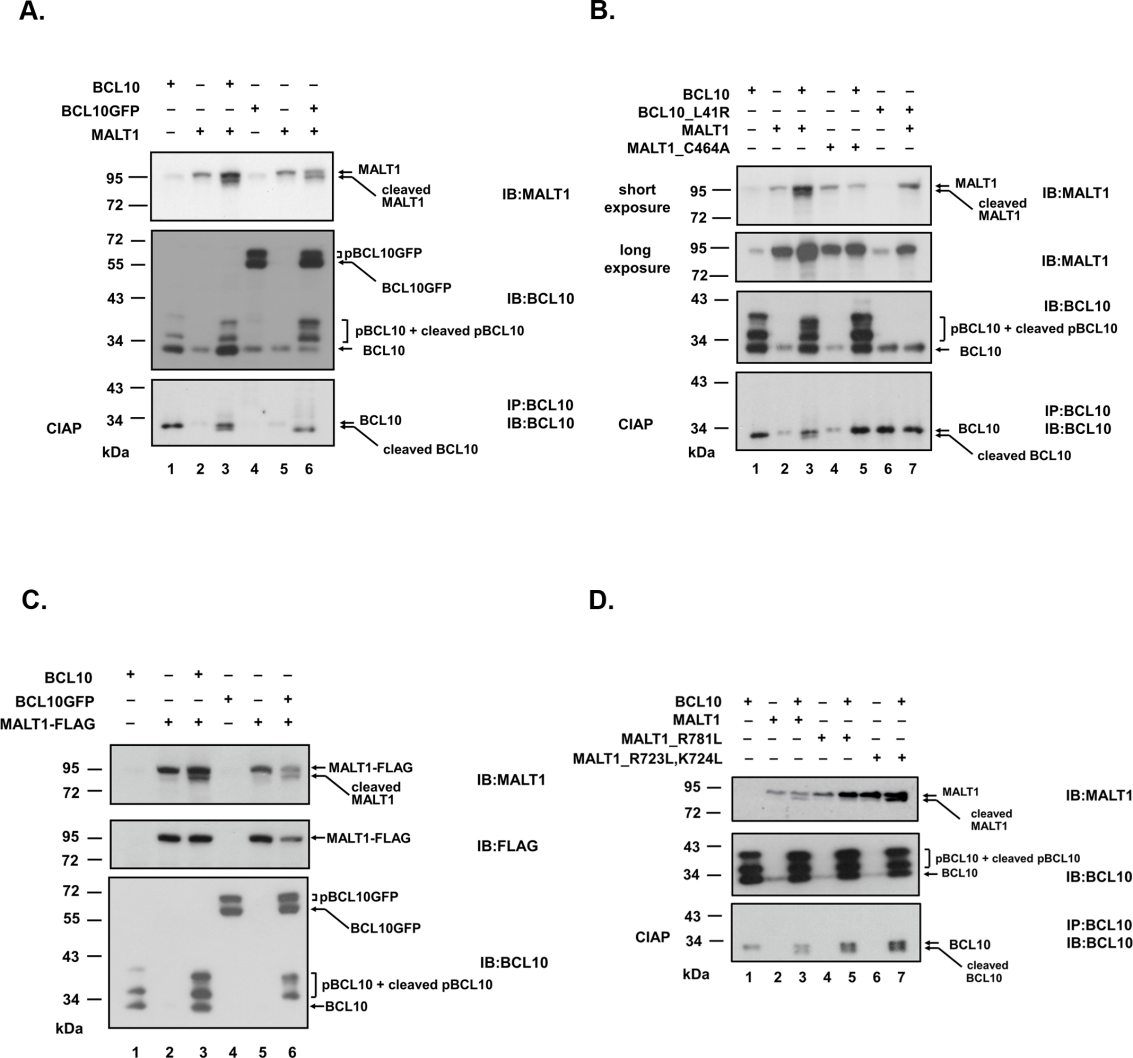
Ectopic expression of BCL10 induces cleavage of MALT1 in HEk293T cells. (A) Lysates of HEk293T cells expressing MALT1 alone or in combination with BCL10 or BCL10GFP were directly analyzed by blotting with anti-MALT1 (sc-46677) or anti-BCL10 (sc-5273) antibodies. BCL10 was phosphorylated intracellularly. The band under 34 kDa was unphosphorylated BCL10. Multiple bands above 34kDa are phosphorylated BCL10 species (pBCL10). Phosphatase (CIAP) treatment of lysates from cells with co-expression of MALT1 revealed the apparent emergence of truncated BCL10 under the unmodified BCL10. (B) Lysates of HEk293T cells expressing MALT1 or catalytically-inactive mutant MALT1_C464A alone or in combination with BCL10 or oligomerization-defective mutant BCL10_L41R [29] were immunoblotted with anti-MALT1 or anti-BCL10 antibodies. (C) Lysates of HEk293T cells expressing MALT1-FLAG (C-terminus FLAG-tagged MALT1) alone or in combination with BCL10 or BCL10GFP were directly analyzed by blotting with anti-MALT1, anti-FLAG or anti-BCL10 antibodies. (D) Lysates of HEk293T cells expressing MALT1, MALT1_R781L, MALT1_R723L,k724L alone or in combination with BCL10 were immunoblotted with anti-MALT1 or anti-BCL10 antibodies. In (A),(B),(D), immunoprecipitation with anti-BCL10 antibody was performed. Immunoprecipitates were treated with Calf Intestine Alkaline Phosphatase (CIAP) before being analyzed with anti-BCL10 antibody to better visualize BCL10 cleavage products. pBCL10: phosphorylated BCL10, pBCL10GFP: phosphorylated BCL10GFP, “cleaved MALT1” indicated “the faster migrating species of MALT1” in the text.

We [27] and others [10,11,15,18,28] showed that overexpression of BCL10 activated MALT1. BCL10 served as an activator and also a substrate of MALT1. BCL10 oligomerized (S2 Fig) and was phosphorylated intracellularly. The appearance of a truncated form of BCL10 (Fig 1A and 1B) revealed the proteolytic activity of MALT1. In addition to BCL10 cleavage, the appearance of a faster migrating species of MALT1 from lysates of cells cotransfected with MALT1 and BCL10 was consistently observed (Fig 1A and 1B). Similar results were shown using BCL10GFP as substituent of BCL10 (Fig 1A). MALT1_C464A, a catalytically-inactive mutant, neither cleaved BCL10 (Fig 1B) nor generated the faster migrating species of MALT1 (Fig 1B). BCL10_L41R, caspase recruitment domain mutant, was previously shown to lose its oligomerization ability [29] (S2 Fig) and fail to activate MALT1 [27]. The faster migrating species of MALT1 was not detected in lysates of cells cotransfected with MALT1 and BCL10_L41R (Fig 1B). These results suggested that MALT1 was processed most likely by itself to a shorter form upon activation by BCL10 in an overexpression system.

FLAG-tag was fused at the C-terminus of MALT1 to assess the possible cleavage site. In the presence of BCL10 or BCL10GFP, MALT1-FLAG was processed to a shorter form as MALT1. Immunoblots with anti-MALT1 antibody revealed the presence of both full length and the faster migrating species of MALT1 (Fig 1C). However, anti-FLAG antibody failed to detect the faster migrating species of MALT1 (Fig 1C). The result indicated that the cleavage site might locate close to the C-terminus of MALT1. MALT1 cleaves its substrates right after an arginine in the P1 position [8], with the exception of LIMA 1a which is cleaved after a lysine [30]. R723, k724 and R781 were mutated to determine the cleavage site. While MALT1_R723L,k724L mutant proteins were processed as wild type MALT1 (Fig 1D), MALT1_R781L mutant proteins lost its ability to be processed (Fig 1D). Both mutant proteins retained their abilities to cleave BCL10 as wild type MALT1. These results indicated that R781 is most likely the self-cleavage site of MALT1. Nucleotide sequence and protein sequence alignment surrounding R781 of MALT1 in different species were performed (S3 Fig). Sequence conservation points to a functional relevance of the cleavage.

### MALT1 cleaves itself *in vitro*

**Fig 2:**
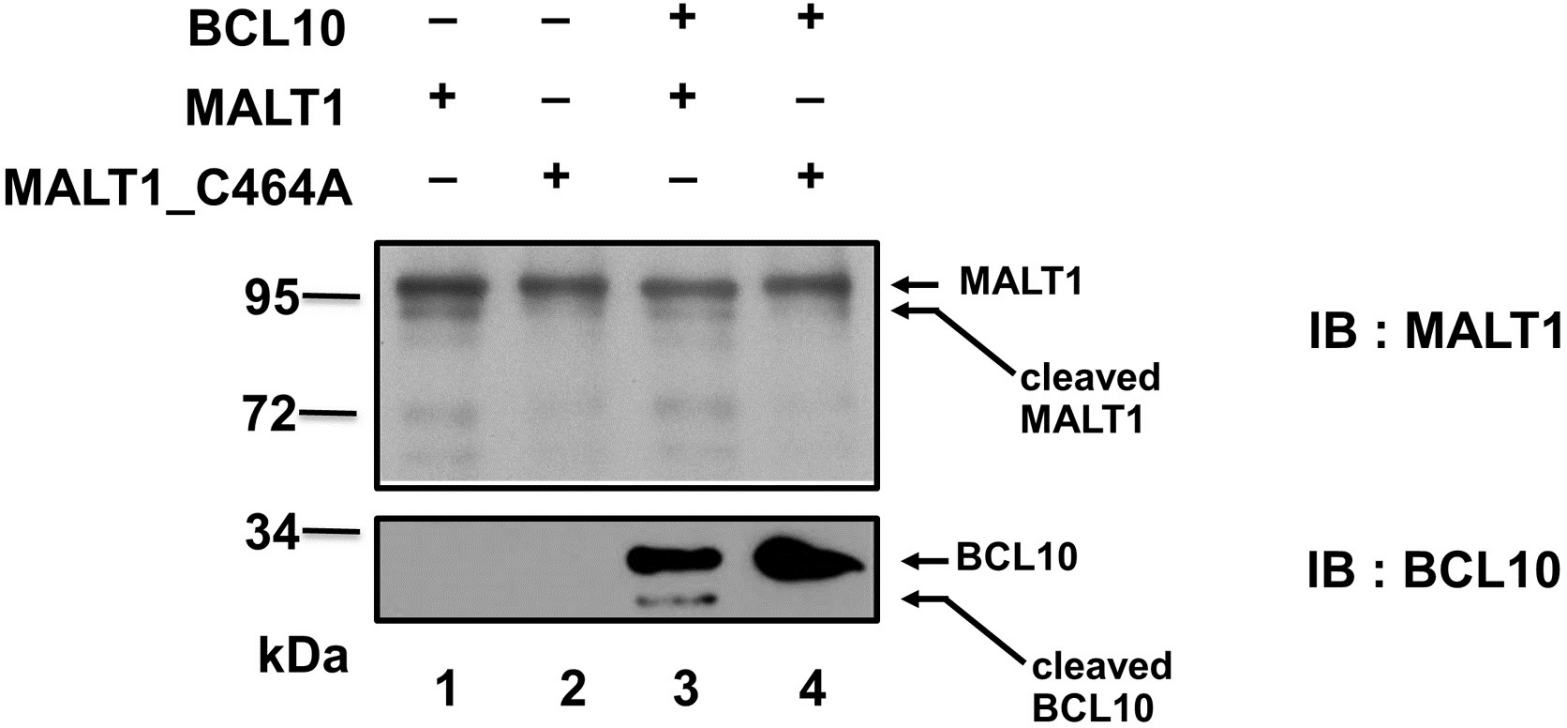
MALT1 autoprocesses itself *in vitro*. His-tagged BCL10, His-tagged MALT1 or MALT1_C464A mutant protein was expressed in *E*. *coli* and purified respectively. 1 μg purified full length MALT1 or catalytic-inactive mutant MALT1_C464A alone or in combination with 50 ng purified BCL10 proteins were incubated for 4 hr at 30°C in 50 mM MES (pH 6.8), 150 mMNaCl, 10% sucrose, 0.1% CHAPS, 10 mM DTT, and 1 M ammonium citrate. The reaction mixtures were resolved by SDS/PAGE, followed by Western blot analysis using anti-MALT1 or anti-BCL10 antibody. Data are representative of three separate experiments.

To further demonstrate a direct self-cleavage of MALT1, we set up an *in vitro* cleavage assay with purified recombinant MALT1 and MALT1_C464A proteins. Purified MALT1 was able to cleave purified BCL10 *in vitro* (Fig 2). Catalytically-inactive MALT1_C464A mutant protein failed to cleave BCL10 (Fig 2). The faster migrating species of MALT1 were present in reactions with catalysis-competent MALT1 (Fig 2) but not with catalytically-inactive MALT1_C464A mutant protein (Fig 2). Since the reaction was set up in the presence of 1mM kosmotropic salt ammonium citrate, self-proteolysis of MALT1 can be demonstrated with or without BCL10. MALT1 was previously shown by Baens *et al*.[15] to process itself at R149, generating p76 and p16 fragment. We also noticed the generation of p76 in the *in vitro* reaction with recombinant MALT1 protein.

### MALT1 undergoes self-cleavage upon activation in T and B lymphocytes

**Fig 3:**
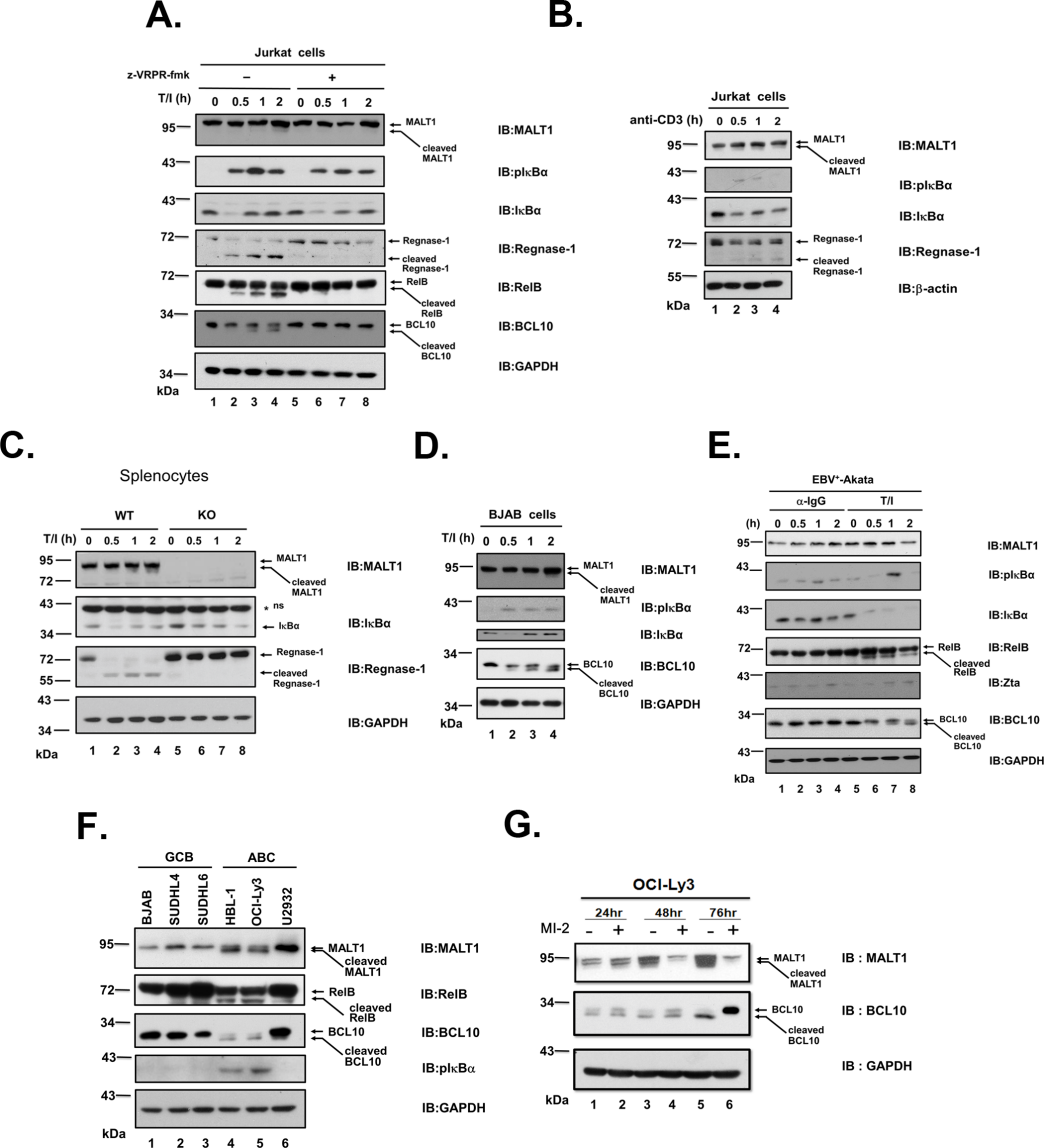
MALT1 undergoes self-cleavage upon activation in T and B lymphocytes. (A) Jurkat T cells were untreated or stimulated with 10 ng/ml TPA and 1 μM ionomycin for the indicated period of times. Cells were pre-treated with 50 μM z-VRPR fmk for 30 mins to inhibit proteolytic activity of MALT1. Lysates were analyzed for NF-κB activation status by blotting with pIκBα and IκBα. Lysates were also analyzed for cleavage of MALT1, Regnase-1, RelB, and BCL10. GAPDH was utilized as loading control. (B) Lysates of Jurkat T cells untreated or stimulated with 10 ng/ml anti-CD3 antibody for the indicated period of times were analyzed. (C) Splenocytes from wild type or MALT1-knockout mice were isolated and stimulated with TPA/ionomycin for the indicated period of times. Lysates were extracted and analyzed for MALT1, IκBα, and Regnase-1. (D) BJAB cells were untreated or stimulated with 10 ng/ml TPA and 1 μM ionomycin for the indicated period of times. Lysates were analyzed for NF-κB activation status by blotting with pIκBα and IκBα. Lysates were also analyzed for cleavage of MALT1 and BCL10. (E) EBV^**+**^-Akata cells were untreated, stimulated with 10 ng/ml TPA and 1 μM ionomycin or with 1% anti-IgG for the indicated period of times. Lysates were analyzed for NF-κB activation status by blotting with pIκBα and IκBα. Lysates were also analyzed for cleavage of MALT1, RelB and BCL10. BCR activation led to activation of EBV in EBV^**+**^-Akata cells. Zta, immediate early antigen of EBV, was analyzed for EBV activation. (F) Lysates of GCB-DLBCL cell lines BJAB, SUDHL-4, SUDHL-6 and ABC-DLBCL cell lines HBL-1, OCI-Ly3, U2932 were analyzed for cleavage of MALT1, RelB, and BCL10. Lysates were analyzed for NF-κB activation status by blotting with pIκBα. (G) Immunoblots analysis of lysates from OCI-Ly3 cells treated with 2 μM MI-2 for 24, 48, and 72 hours was shown. Data are representative of three separate experiments.

T or B lymphocytes were utilized to investigate whether proteolysis of endogenous MALT1 occurred. The faster migrating species of MALT1 appeared along with the appearance of cleaved BCL10, RelB, and Regnase-1 in Jurkat cells stimulated with TPA plus ionomycin (Fig 3A). The cleavage events were prevented by treatment of cells with MALT1 protease inhibitor z-VRPR-fmk (Fig 3A). The cleavage of MALT1 was also found in Jurkat cells treated with anti-CD3 antibody (Fig 3B). Proteolysis of MALT1 occurred in isolated mouse splenocytes upon stimulation with TPA/ionomycin (Fig 3C). Stimulation of BJAB cells with TPA and ionomycin led to similar results (Fig 3D). BCR activation by either cross-linking surface IgG with anti-IgG or TPA/ionomycin in EBV^**+**^-Akata cells did not result in the prominent cleavage of MALT1 (Fig 3E). The human activated B-cell like diffuse large B-cell lymphoma (ABC-DLBCL) cells are characterized by chronic active BCR signaling [19,31]. These cells were shown previously to have constant NF-κB activation and MALT1 protease activity [21,22]. Proteolysis of MALT1 along with cleavage of BCL10 and RelB was detected in the ABC-DLBCL cell lines HBL-1, OCI-Ly3, but not in the GCB-DLBCL cell lines BJAB, SUDHL-4 and SUDHL-6 (Fig 3F). Upon treatment of OCI-Ly3 cells with irreversible MALT1 protease inhibitor MI-2, the levels of full length BCL10 and MALT1 significantly increased (Fig 3G). All these results indicated that endogenous MALT1 undergoes cleavage upon activation of antigen-receptor pathways in a physiology-relevant condition.

### Truncation of MALT1 at R781 attenuates its NF-κB activation ability

**Fig 4:**
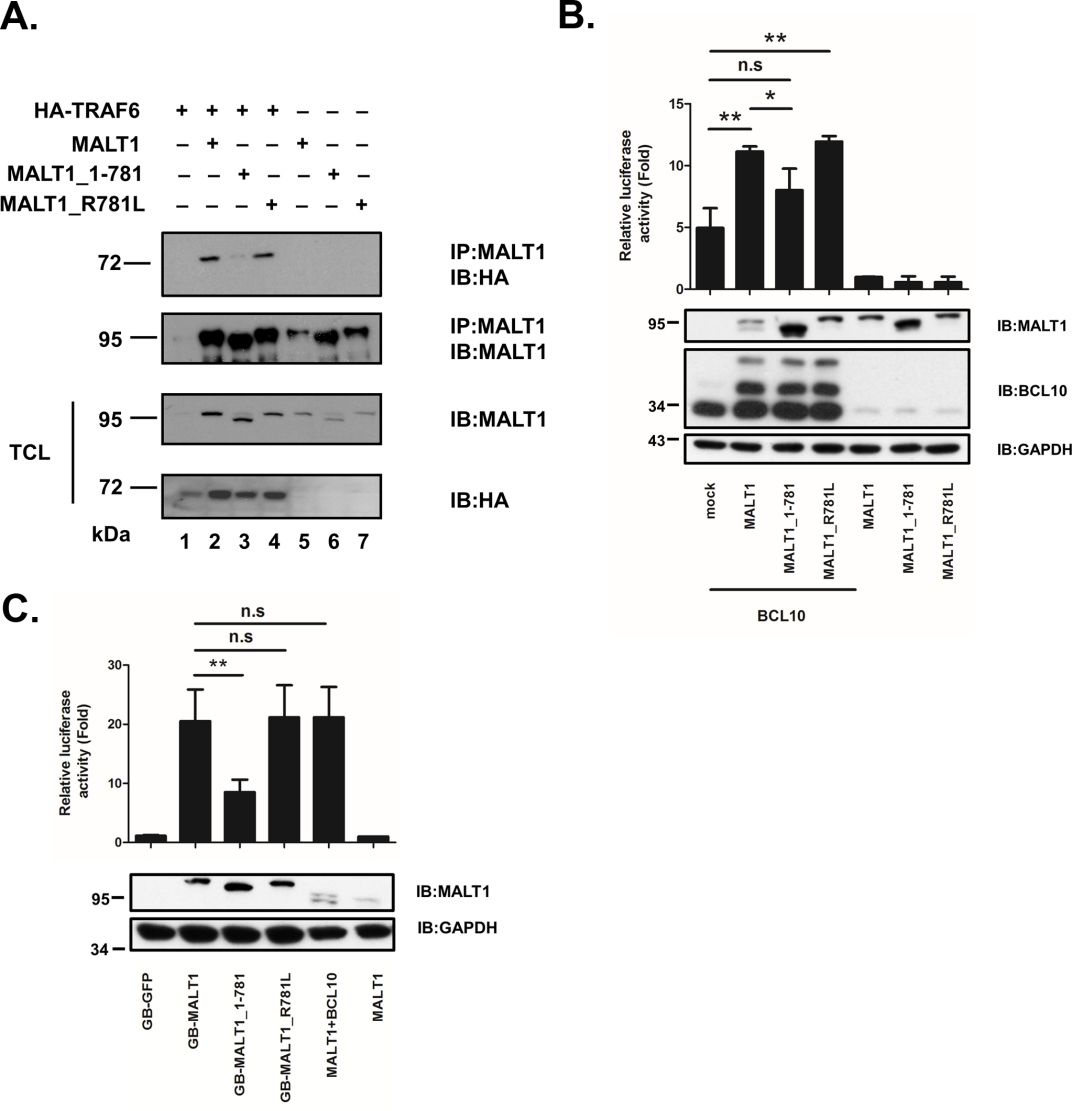
C-terminus-truncated MALT1 shows attenuated NF-κB activation ability. (A) MALT1, MALT1_1–781, or MALT1_R781L was expressed alone or in combination with HA-tagged TRAF6 in HEk293T cells. Lysates were immunoprecipitated with anti-MALT1 antibody then immunoblotted with anti-HA or anti-MALT1 antibody. Total cell lysates (TCL) were directly analyzed for expression of MALT1 or TRAF6 with anti-MALT1 or anti-HA antibody. (B) NF-κB reporter assay of HEk 293T cells expressing MALT1, MALT1_1–781, or MALT1_R781L alone or in combination with BCL10 was performed. (C) NF-κB reporter assay of HEk 293T cells expressing GB-GFP, GB-MALT1, GB-MALT1_1–781, or GB-MALT1_R781L was performed. NF-κB-dependent luciferase activity is shown as fold induction of vector-transfected cells and represents the mean +/- S.D. (n = 3).*:*P*<0.05, **:*P*<0.01, n.s.: not significant.

MALT1 contains two TRAF6 binding sites (P_651_E**E**TGSA_657_ and P_804_V**E**TTD_809_) [5]. Mutation of these sites affects its ability to interact with TRAF6 and hence impairs the Ikk activation ability [5]. Cleavage at R781 removes one key TRAF6 binding site. Co-immunoprecipitation and NF-κB reporter assay were set up to investigate whether C-terminus processing of MALT1 at R781 has a role in the binding with TRAF6 and NF-κB signaling. Immunoprecipitation experiments showed that HA-TRAF6 coprecipitated with full length MALT1 and MALT1_R781L (Fig 4A). MALT1_1–781, losing a major TRAF6 binding site, was not able to interact with TRAF6 as well (Fig 4A). Full length MALT1 alone did not activate NF-κB reporter in HEk293T cells. However, MALT1 potentiated the ability of BCL10 to stimulate NF-κB reporter activity. MALT1_R781L retained its ability to stimulate BCL10 as well. The ability of MALT1_1–781 mutant protein to potentiate BCL10 to activate NF-κB was impaired (Fig 4B). Fusion of bacterial gyrase B dimerization domain allows the formation of MALT1 oligomers and has been shown to activate NF-κB activity [5,11]. We replaced the death domain of MALT1 with gyrase B dimerization domain, generating GB-MALT1 fusion protein (S1 Fig, S1 Text). GB-MALT1 and GB-MALT1_R781L potently activated NF-κB reporter as expected (Fig 4C). Unlike GB-MALT1 and GB-MALT1_R781L, GB-MALT1_1–781 exhibited two fold reduction in NF-κB activation ability. Collectively, these data indicated that cleavage of MALT1 at R781 weakened the interaction with TRAF6 and attenuated NF-κB signaling pathway.

### Truncation of MALT1 at R781 affected NF-κB activity in Jurkat T cells

**Fig 5:**
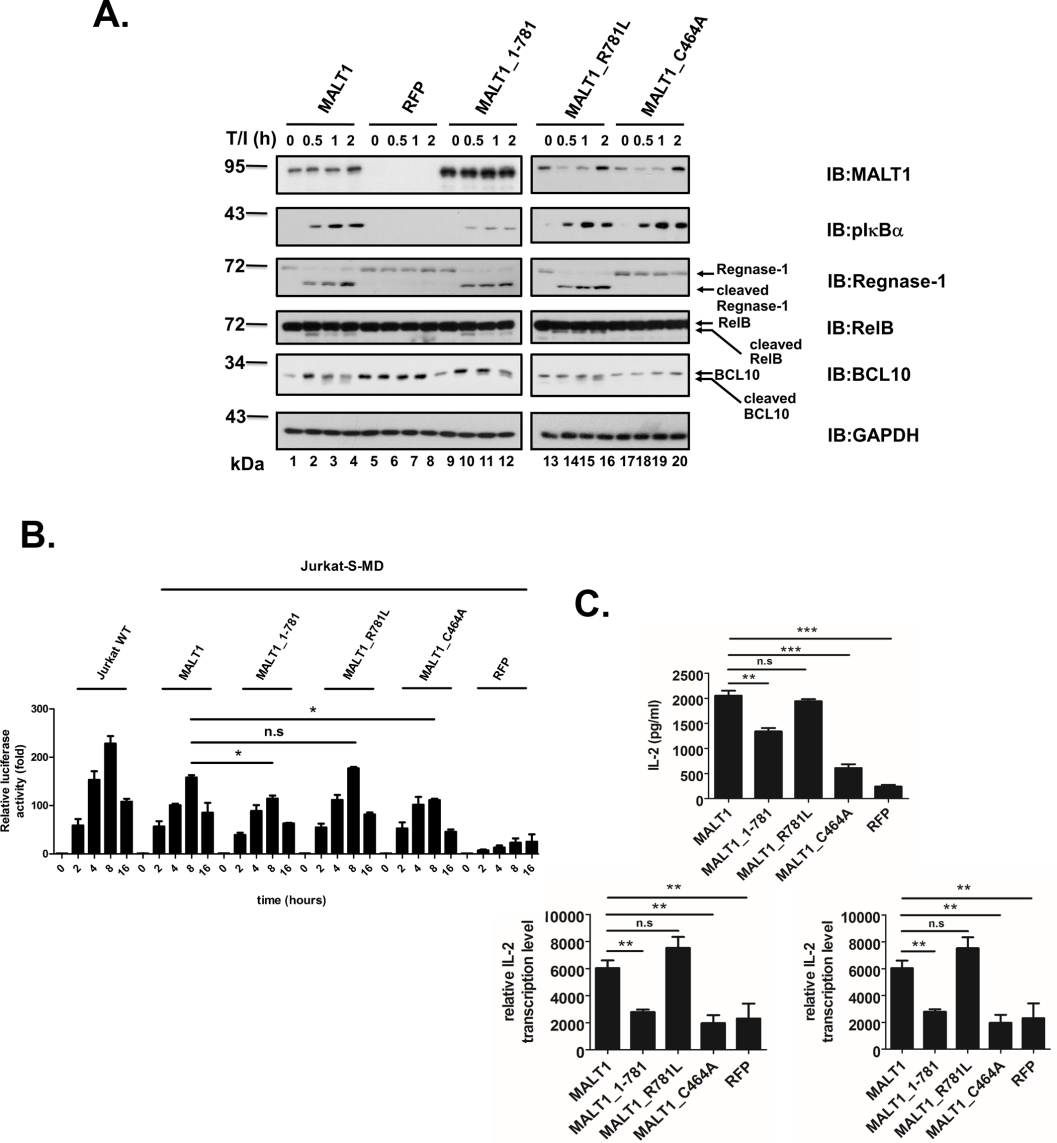
Protelytic processing of MALT1 at R781 impairs activation-induced NF-κB activity in Jurkat T cells. MALT1-knocked-down Jurkat cells (Jurkat-S-MD) were transduced with lentiviral expression constructs of RFP (control), wild type MALT1 or the MALT1_1–781, MALT1_R781L, MALT1_C464A mutant. (A) Immunoblot analysis of lysates of these cells untreated or stimulated with TPA/ionomycin for indicated times with indicated antibodies. (B) NF-κB reporter luciferase assay of cells electroporated with NF-κB reporter construct. (C) IL-2 concentration in supernatants of indicated Jurkat T cells stimulated for 8 hrs with TPA/ionomycin were measured. IL-2 and IFN-γ transcript levels were determined via qRT-PCR *:*P*<0.05, **:*P*<0.01, ***:*P*<0.001, n.s.: not significant.

To assess the effect of cleavage of MALT1 at R781 on T cell activation, MALT1-knocked-down Jurkat T cells with expression of wild type MALT1, truncated mutant MALT1_1–781, cleavage-defective mutant MALT1_R781L and catalytically-inactive mutant MALT1_C464A were generated. Phosphorylation level of IκBα was utilized to monitor the scaffolding ability of MALT1 after stimulation of these cells with TPA plus ionomycin. Proteolytic activity of MALT1 and variants was monitored by checking substrates such as Regnase-1, RelB, and BCL10. knocking down of MALT1 expression impaired both scaffolding and proteolytic function of MALT1 (Fig 5A). Catalytically-inactive mutant MALT1_C464A retained its scaffolding ability but lost its proteolytic activity. While the induced-phosphorylation of IκBα was not affected by cleavage-defective mutant MALT1_R781L the level of p-IκBα was reduced in stimulated cells with expression of truncated mutant MALT1_1–781. In contrast to MALT1_C464A, MALT1_1–781 and MALT1_R781L retained their ability to process Regnase-1, RelB, and BCL10 as wild type MALT1. The capacity of wild type and various MALT1 mutants to reconstitute the activation of NF-κB reporter in Jurkat cells with knocked-down expression of MALT1 was also examined. Inactivation of catalytic activity or truncation at R781 on MALT1 led to partially impaired activation of NF-κB reporter luciferase activity (Fig 5B). Stimulation-induced IL-2 secretion of MALT1_1–781 expressing cells was reduced by 54% as compared to a 68% reduction in MALT1_C464A expressing cells. qRT-PCR analysis showed also defects in the up-regulation of IL-2 and IFN-γ mRNA levels in cells with expression of MALT1_1–781 as well as MALT1_C464A (Fig 5C). These data indicated that self-cleavage of MALT1 at R781 dampened NF-κB signaling upon TCR activation.

### Proteolysis of MALT1 at R781 is required for the survival of ABC-DLBCL cells

**Fig 6:**
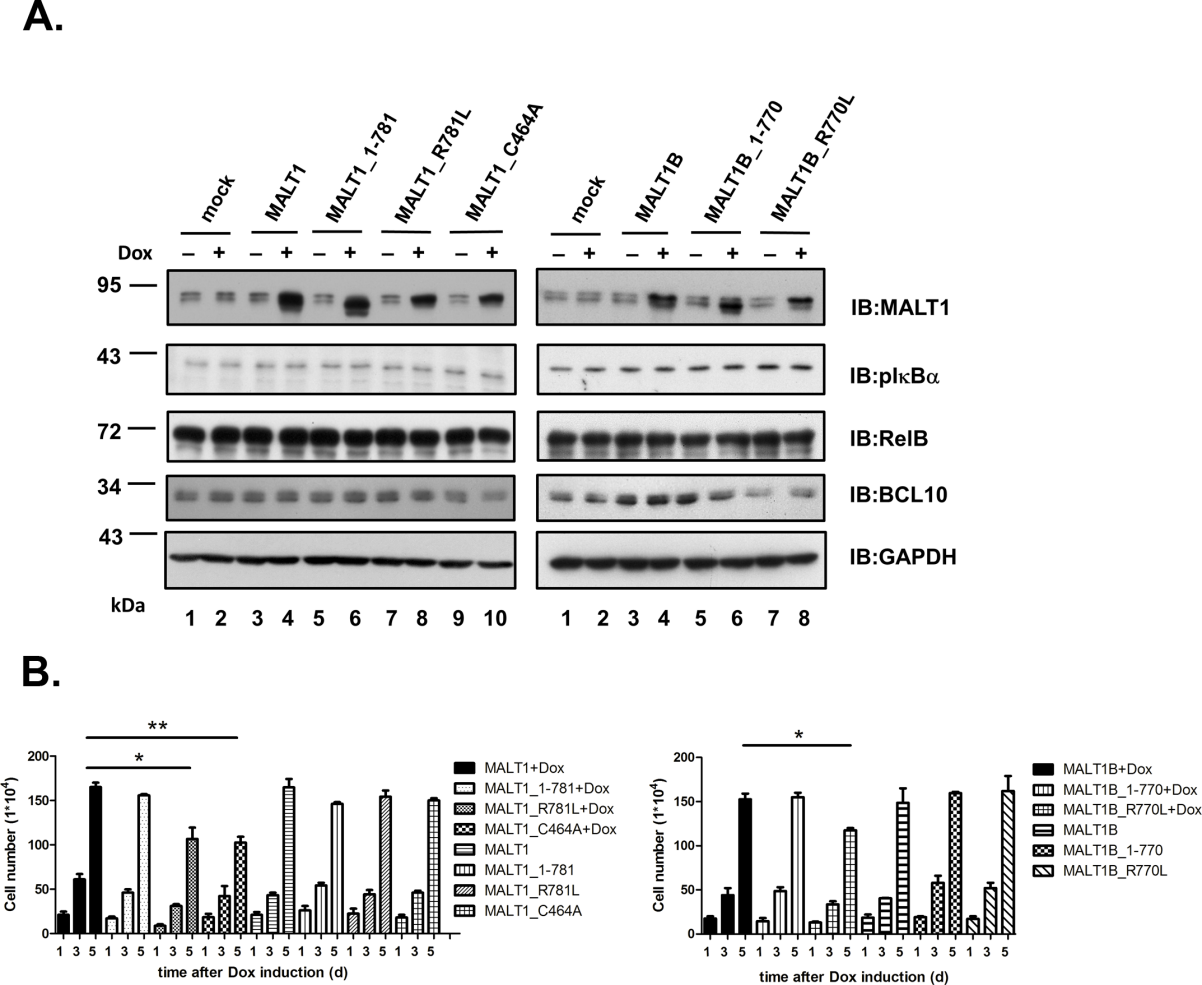
Cleavage-defective MALT1_R781L as well as catalytically-inactive MALT1_C464A affects the growth of HBL-1 cells. HBL-1 cells were lentivirally transduced with doxycycline-inducible expression vectors for MALT1, MALT1_1–781, MALT1_R781L, MALT1_C464A, MALT1B, MALT1B_1–770, or MALT1B_R770L. (A) Expression levels of endogenous and various transduced forms of MALT1 at 4 days after the addition of doxycycline (+) or not (-) were shown. Lysates were analyzed for NF-κB activation status by blotting with pIκBα. Lysates were also analyzed for cleavage RelB and BCL10. (B) Viable cells were counted using trypan blue exclusion assays at the indicated time point in the absence or presence of doxycycline. Cells were set up in triplicate for each experiment. Shown is the mean +/- S.D. from one representative of three independent experiments. *:*P*<0.05, **:*P*<0.01.

To explore the biological effect of cleavage of MALT1 at R781 in ABC-DLBCL cells, HBL-1 cells were transduced with doxycycline inducible expression vectors for MALT1, MALT1_1–781, MALT1_R781L, and MALT1_C464A. Inhibition of MALT1 protease activity affects the growth and survival of ABC-DLBCL cells [21–24]. Transduction with catalytically inactive MALT1_C464A caused a clear reduction in cell viability (Fig 6B) as shown previously by Hailfinger *et al*. [22]. While truncated MALT1_1–781 had no effect, induced expression of cleavage-defective MALT1_R781L reduced the viability of HBL-1 cells (Fig 6B). HBL-1, ABC-DLBCL cells with chronic active BCR signaling, exhibited higher expression level of pIκBα and cleavage of MALT1, RelB and BCL10 as compared to other GCB-DLBCL cells (Fig 3F). Additionally-induced expression of MALT1 and variants did not affect the status of pIκBα and cleavage of RelB and BCL10 in HBL-1 cells (Fig 6A). MALT1B (S1 Fig), an alternative splicing variant of MALT1 with shortage of 11 amino acids encoded by exon 7, was also examined. MALT1B was shown previously to exhibit a slightly weaker NF-κB activation ability in T cells [32]. While MALT1B and MALT1B_1–770 had no effect, induced expression of MALT1B_R770L reduced the viability of HBL-1 cells as MALT1_R781L. Western blot analysis of pIκBα, RelB and BCL10 in HBL-1 cells transduced with MALT1B (813 aa) led to similar results as those with MALT1 (824 aa).

## Discussion

In this study, paracaspase MALT1 was demonstrated to auto-cleave itself both *in vivo* and *in vitro*. The cleavage site was identified to be R781 near the C terminus of MALT1. Cleavage at this site removes a major TRAF6 binding site (P_804_V**E**TTD_809_). Truncated MALT1_1–781 exhibited weakened ability to interact with TRAF6 and attenuated ability to activate NF-κB reporter gene. Ginster *et al*.[25] identified the same auto-cleavage site using either activated CARD11_L244P in combination with BCL10 or TRAF6 alone. The capacity of the C-terminus truncated MALT1 variant (MALT1_1–781) to activate NF-κB reporter gene was as well as the full length MALT1 in the presence of activated CARD11_L244P [25]. Our studies by co-transfection with BCL10 or fusion with gyrase B dimerization domain indicated that C-terminus truncation of MALT1 reduced its NF-κB activation ability (Fig 4). It is possible that the subtle difference in NF-κB activation ability of full length and C-terminus truncated MALT1 cannot be revealed in the strong NF-κB activation setting possessed by activated CARD11_L244P. Expression of MALT1_1–781 in MALT1-knocked-down-Jurkat T cells impaired IκBα phosphorylation and attenuated expression of the NF-κB targets IL-2 and IFN-γ (Fig 5) in this study. Our results strengthened the notion that auto-cleavage of MALT1 at R781 jeopardized its NF-κB activation ability in activated T cell.

MALT1 directs cleavage on several negative regulators such as A20 [11] and RelB [12] important in NF-κB signaling. In addition, Regnase-1, Roquin-1 and-2 that destabilize subsets of NF-κB-dependent mRNAs, are processed and inactivated by MALT1 [13,14]. Hence, the proteolytic activity of MALT1 was generally believed to optimize NF-κB activation and cellular responses. The identification of E3 ubiquitin ligase HOIL1, a positive regulator of the linear ubiquitin chain assembly complex (LUBAC), as a substrate of MALT1 provides the first evidence for a negative role of MALT1-mediated cleavage in NF-κB signaling [16,18]. Auto-cleavage of MALT1 at R781 offered another support for MALT1-mediated cleavage in negative regulation of NF-κB signaling as proposed on MALT1-mediated cleavage of HOIL1.

Cleavage of negative regulators of NF-κB signaling pathway such as RelB and Regnase-1 were observed as early as 0.5 hrs after stimulation in T cells or B cells. However, MALT1 itself is cleaved at 1 or 2 hours after stimulation in T cells or B cells. We proposed that MALT1 mediated cleavage of RelB or Regnase-1 first to promote activation, then triggered cleavage at R781 of itself to prevent further activation via a negative feedback mechanism.

MALT1 was once reported to auto-process itself at R149 [15]. The cleavage at R149 can be monitored by the production of p76 and p16. In the study by Baens *et al*., the cleavage occurred 30 minutes after activation in lymphocytes and was demonstrated to be essential for NF-κB-dependent gene transcription. The p76 MALT1 fragment generated by cleavage at R149 was demonstrated to be labile in the presence of TRAF6 [25]. Even in the presence of proteasome inhibitor MG-132, cleavage at R149 was barely detectable in the study by Ginster *et al*. Cleavage at R781 was dominant under these conditions. In our study, p76 could only be detected in the *in vitro* cleavage assay, not in the lymphocytes activated with TPA/ionomycin or anti-CD3 (Fig 3C) nor in ABC-DLBCL cells (Fig 3D). Cleavage at R781 was detectable but marginal after stimulation in lymphocytes (Fig 3A, 3B, 3C and 3D). In Jurkat T cells upon activation by TPA/ionomycin, MALT1_R781L shared equal capacity with wild type MALT1 in upregulation of the NF-κB targets IL-2 and IFN-γ (Fig 5). These results raised an issue as how much auto-cleavage of MALT1 at R781 contributed to the regulation of NF-κB signaling in activated T-lymphocytes. Cleavage of MALT1 was marginal in BJAB cells stimulated with TPA/ionomycin (Fig 3D). Cleavage of MALT1 was barely detectable in Akata cells with either TPA/ionomycin or anti-IgG cross linking (Fig 3E). However, cleavage of MALT1 was evident in ABC-DLBCL cells such as OCI-Ly3, HBL-1 (Fig 3F and 3G). Nearly 50% of MALT1 proteins were processed. In addition to BCR activation, additional events must have involved in the apparent cleavage of MALT1 itself in HBL-1 or OCI-Ly3 cells. Future studies are required to dissect the conditions for cleavage of MALT1 at R781 in different cell types.

ABC-DLBCL is characterized by constitutive NF-κB activity and requires signals from CARD11, BCL10, and MALT1 for survival [19]. Inhibition of the MALT1 proteolytic activity with paracaspase inhibitor affected the growth and survival of ABC-DLBCL cell lines [21,22]. The phenomenon was correlated with decreased expression of NF-κB activation signature genes. Transduction with catalytically-inactive MALT1 (MALT1_C464A) into ABC-DLBCL cells caused reduction of cell viability and an increase in apoptotic cells [22]. In analogy with paracaspase inhibitor, catalytically-inactive MALT1_C464A exerted these effects possibly through down-regulation of NF-κB activity. Indeed, MALT1 mutated in the predicted active site C464 showed a reduction in their capacity to activate NF-κB reporter gene in transfected cells [28,33]. Overexpression of catalytically inactive MALT1_C464A reduced expression of several NF-κB dependent transcripts in lymphocytes upon activation [11,34–36]. Induced expression of MALT1 and variants were performed in HBL-1 cells with activated endogenous MALT1 and balanced NF-κB activity. Both MALT1_R781L and MALT1_C464A exerted dominant-negative effects over endogenous MALT1 and affected the viability of HBL-1 cells. MALT1 is known to control NF-kB signaling through both its scaffold to recruit TRAF6 and protease function. Proteolytic activity of MALT1 played a critical role in the growth and survival of ABC-DLBCL cells [21,22]. MALT1_C464A might affect the growth and survival of HBL-1 cell through defect in proteolytic activity of MALT1. MALT1_1–781 with attenuated ability to recruit TRAF6 still retained its proteolytic activity which was sufficient for the growth of HBL-1 cells. MALT1_R781L retained intact proteolytic activity to process negative regulators and held ability to recruit TRAF6 as scaffold. It is tempting to speculate that negative regulation through cleavage of MALT1 at R781 was impaired in HBL-1 cells with expression of MALT1_R781L. The outcome of defect in negative regulation resulted in unrestricted NF-κB activation and unbalanced growth. Status of pIκBα, the proximal signaling molecule in antigen-receptor-activated NF-κB pathway, was examimed in cells with induced expression of MALT1, MALT1-1-781, MALT1_R781L or MALT1_C464A and found to be the same. How exactly cleavage-defective MALT1_R781L mutant protein affected the growth of ABC-DLBCL cells awaits for determination.

knock-in (kI) mice with expression of catalytically inactive MALT1 mutant developed multi-organ inflammation [26, 34–36]. Excessive production of NF-κB downstream target IFN-γ in effector lymphocytes was demonstrated to mediate the effect. Exact mechanism by which the catalytically inactive MALT1 mutant dysregulates the expression of IFN-γ is not yet addressed. Catalytically inactive MALT1 lost the proteolytic ability on multiple substrates including itself. Reduced negative feedback regulation of NF-κB signaling caused by the absence of MALT1-mediated cleavage on itself at R781 might possibly contribute to this phenotype. kI mice expressing a cleavage-defective MALT1 mutant (MALT1_R781L) will be helpful on elucidating the issue.

As increasing substrates of MALT1 were identified, the proteolytic activity of MALT1 could play either a positive or a negative regulator in NF-κB signaling. The interplay between these two roles needs further investigation.

## Conclusions

We offered evidence that MALT1 cleaved itself after R781 both *in vitro* and *in vivo*. The cleavage was evident in ABC-DLBCL cells and played a role in the survival of ABC-DLBCL cells.

## Supporting information

S1 Fig Schematic representation of MALT1 and variants. Different domains of MALT1 are shown. DD; Death Domain, Ig: Immunoglobulin-like domain, CLD: Caspase Like Domain, T6BM: TRAF 6 Binding Motif, GBDiD: Gyrase B Dimerization Domain. The catalytic cysteine residue and the autocleavage site are also shown in red.
(TIF)

S2 Fig Fluorescence micrographs of BCL10GFP and BCL10^L41R^GFP. BCL10 oligomerizes and exhibits a discrete cytoplasmic filaments when overexpressed. BCL10_L41R with mutation in caspase recruitment domain exhibits a diffused pattern and fails to form filaments. Formation of discrete cytoplasmic filaments was utilized as oligomerization indicator. Phosphorylation of BCL10 correlates with filament formation.
(DOCX)

S3 Fig (A) Nucleotide sequence and (B) protein sequence alignment surrounding R781 of MALT1 in different species.
(TIF)

S1 Text Supplemental experimental procedures. (DOCX)

## References

[1] Demeyer A, Staal J, Beyaert R. Targeting MALT1 Proteolytic Activity in Immunity, Inflammation and Disease: Good or Bad? Trends Mol Med. 2016;22:135–50. doi: 10.1016/j.molmed.2015.12.004 26787500 10.1016/j.molmed.2015.12.004

[2] Jaworski M, Thome M. The paracaspase MALT1: biological function and potential for therapeutic inhibition. Cell Mol Life Sci. 2016;73:459–73. doi: 10.1007/s00018-015-2059-z 26507244 10.1007/s00018-015-2059-z PMC4713714

[3] Wegener E, krappmann D. CARD-Bcl10-Malt1 signalosomes: missing link to NF-kappaB.Sci STkE. 2007;384:pe21. 10.1126/stke.3842007pe21 17473310

[4] Qiao Q, Yang C, Zheng C, Fontán L, David L, Yu X, et al Structural architecture of the CARMA1/Bcl10/MALT1 signalosome: nucleation-induced filamentous assembly. Mol Cell 2013;51:766–79. doi: 10.1016/j.molcel.2013.08.032 24074955 10.1016/j.molcel.2013.08.032 PMC3929958

[5] Sun L, Deng L, Ea Ck, Xia ZP, Chen ZJ. The TRAF6 ubiquitin ligase and TAk1 kinase mediate Ikk activation by BCL10 and MALT1 in T lymphocytes. Mol Cell 2004;14: 289–301. 15125833 10.1016/s1097-2765(04)00236-9

[6] Wu CJ, Ashwell JD. NEMO recognition of ubiquitinated BCL10 is required for T cell receptor-mediated NF-κB activation. Proc Natl Acad Sci USA. 2008;105:3023–28. doi: 10.1073/pnas.0712313105 18287044 10.1073/pnas.0712313105 PMC2268578

[7] Oeckinghaus A, Wegener E, Welteke V, Ferch U, Arslan SC, Ruland J, et al MALT1 ubiquitination triggers NF-κB signaling upon T-cell activation. EMBO J. 2007;26:4634–45. doi: 10.1038/sj.emboj.7601897 17948050 10.1038/sj.emboj.7601897 PMC2080808

[8] Hachmann J, Snipas SJ, van Raam BJ, Cancino EM, Houlihan EJ, Poreba M, et al Mechanism and specificity of the human paracaspase MALT1. Biochem J. 2012;443:287–95. doi: 10.1042/BJ20120035 22309193 10.1042/BJ20120035 PMC3304489

[9] Wiesmann C, Leder L, Blank J, Bernardi A, Melkko S, Decock A, et al Structural determinants of MALT1 protease activity. J Mol Biol. 2012;419:4–21. doi: 10.1016/j.jmb.2012.02.018 22366302 10.1016/j.jmb.2012.02.018

[10] Rebeaud F, Hailfinger S, Posevitz-Fejfar A, Tapernoux M, Moser R, Rueda D, et al The proteolytic activity of the paracaspase MALT1 is key in T cell activation. Nat Immunol. 2008;9:272–81. doi: 10.1038/ni1568 18264101 10.1038/ni1568

[11] Coornaert B, Baens M, Heyninck k, Bekaert T, Haegman M, Staal J, et al T cell antigen receptor stimulation induces MALT1 paracaspase-mediated cleavage of the NF-κB inhibitor A20. Nat Immunol. 2008;9: 263–71. doi: 10.1038/ni1561 18223652 10.1038/ni1561

[12] Hailfinger S, Nogai H, Pelzer C, Jaworski M, Cabalzar k, Charton JE, et al Malt1-dependent RelB cleavage promotes canonical NF-κB activation in lymphocytes and lymphoma cell lines. Proc Natl Acad Sci USA. 2011;108:14596–601. doi: 10.1073/pnas.1105020108 21873235 10.1073/pnas.1105020108 PMC3167514

[13] Uehata T, Iwasaki H, Vandenbon A, Matsushita k, Hernandez-Cuellar E, kuniyoshi k, et al Malt1-induced cleavage of regnase-1 in CD4(+) helper T cells regulates immune activation. Cell 2013;153:1036–49. doi: 10.1016/j.cell.2013.04.034 23706741 10.1016/j.cell.2013.04.034

[14] Jeltsch kM, Hu D, Brenner S, Zöller J, Heinz GA, Nagel D, et al Cleavage of roquin and regnase-1 by the paracaspase MALT1 releases their cooperatively repressed targets to promote T(H)17 differentiation. Nat Immunol. 2014;15:1079–89. doi: 10.1038/ni.3008 25282160 10.1038/ni.3008

[15] Baens M, Bonsignore L, Somers R, Vanderheydt C, Weeks SD, Gunnarsson J, et al MALT1 auto-proteolysis is essential for NF-κB-dependent gene transcription in activated lymphocytes. PLoSONE 2014;9: e103774. 10.1371/journal.pone.0103774 PMC4126661 25105596

[16] klein T, Fung SY, Renner F, Blank MA, Dufour A, kang S, et al The paracaspase MALT1 cleaves HOIL1 reducing linear ubiquitination by LUBAC to dampen-lymphocyte NF-κB signalling. Nat Commun. 2015;6:8777 doi: 10.1038/ncomms9777 26525107 10.1038/ncomms9777 PMC4659944

[17] Douanne T, Gavard J, Bidère N. The paracaspase MALT1 cleaves the LUBAC subunit HOIL1 during antigen receptor signaling. J Cell Sci. 2016;129:1775–80. doi: 10.1242/jcs.185025 27006117 10.1242/jcs.185025

[18] Elton L, Carpentier I, Staal J, Driege Y, Haegman M, Beyaert R. MALT1 cleaves the E3 ubiquitin ligase HOIL-1 in activated T cells, generating a dominant negative inhibitor of LUBAC-induced NF-κB signaling. FEBS J. 2016;283:403–12. doi: 10.1111/febs.13597 26573773 10.1111/febs.13597

[19] Ngo VN, Davis RE, Lamy L, Yu X, Zhao H, Lenz G, et al A loss-of-function RNA interference screen for molecular targets in cancer. Nature 2006;44:106–10. 10.1038/nature04687 16572121

[20] Davis RE, Brown kD, Siebenlist U, Staudt LM. Constitutive nuclear factor kappaB activity is required for survival of activated B cell-like diffuse large B cell lymphoma cells. J Exp Med. 2001;194:1861–74. 11748286 10.1084/jem.194.12.1861 PMC2193582

[21] Ferch U, kloo B, Gewies A, Pfänder V, Düwel M, Peschel C, et al Inhibition of MALT1 protease activity is selectively toxic for activated B cell-like diffuse large B cell lymphoma cells. J Exp Med. 2009;206:2313–20. doi: 10.1084/jem.20091167 19841089 10.1084/jem.20091167 PMC2768866

[22] Hailfinger S, Lenz G, Ngo V, Posvitz-Fejfar A, Rebeaud F, Guzzardi M, et al Essential role of MALT1 protease activity in activated B cell-like diffuse large B-cell lymphoma. Proc Natl Acad Sci USA. 2009;106:19946–51. doi: 10.1073/pnas.0907511106 19897720 10.1073/pnas.0907511106 PMC2785272

[23] Fontan L, Yang C, kabaleeswaran V, Volpon L, Osborne MJ, Beltran E, et al MALT1 small molecule inhibitors specifically suppress ABC-DLBCL in vitro and in vivo. Cancer Cell 2012;22:812–24. doi: 10.1016/j.ccr.2012.11.003 23238016 10.1016/j.ccr.2012.11.003 PMC3984478

[24] Nagel D, Spranger S, Vincendeau M, Grau M, Raffegerst S, kloo B, et al Pharmacologic inhibition of MALT1 protease by phenothiazines as a therapeutic approach for the treatment of aggressive ABC-DLBCL. Cancer Cell 2012;22:825–37. doi: 10.1016/j.ccr.2012.11.002 23238017 10.1016/j.ccr.2012.11.002

[25] Ginster S, Bardet M, Unterreiner A, Malinverni C, Renner F, Lam S, et al Two antagonistic MALT1 auto-cleavage mechanisms reveal a role for TRAF6 to unleash MALT1 activation. PLoSONE 2017;12:e0169026. 10.1371/journal.pone.0169026 PMC5214165 28052131

[26] Jaworski M, Marsland BJ, Gehrig J, Held W, Favre S, Luther SA, et al Malt1 protease inactivation efficiently dampens immune responses but causes spontaneous autoimmunity. EMBO J. 2014;33:2765–81. doi: 10.15252/embj.201488987 25319413 10.15252/embj.201488987 PMC4282555

[27] Jou SY, Chang CC, Wu CH, Chen MR, Tsai CH, Chuang WH, et al BCL10GFP fusion protein as a substrate for analysis of determinants required for Mucosa-Associated Lymphoid Tissue 1 (MALT1)-mediated cleavage. J Biomed Sci. 2012;19:85 doi: 10.1186/1423-0127-19-85 23035874 10.1186/1423-0127-19-85 PMC3500650

[28] Lucas PC, Yonezumi M, Inohara N, McAllister-Lucas LM, Abazeed ME, Chen FF, et al BCL10 and MALT1, independent targets of chromosomal translocation in malt lymphoma, cooperate in a novel NF-κB signaling pathway. J Biol Chem. 2001;276:19012–19. doi: 10.1074/jbc.M009984200 11262391 10.1074/jbc.M009984200

[29] Guiet C, Vito P. Caspase recruitment domain (CARD)-dependent cytoplasmic filaments mediate bcl10-induced NF-kappaB activation. J Cell Biol. 2000;148:1131–40. 10725326 10.1083/jcb.148.6.1131 PMC2174297

[30] Nie Z, Du MQ, McAllister-Lucas LM, Lucas PC, Bailey NG, Hogaboam CM, et al Conversion of the LIMA1 tumour suppressor into an oncogenic LMO-like protein by API2-MALT1 in MALT lymphoma. Nat Commun. 2015;6:5908 doi: 10.1038/ncomms6908 25569716 10.1038/ncomms6908

[31] Lenz G, Davis RE, Ngo VN, Lam L, George TC, Wright GW, et al Oncogenic CARD11 mutations in human diffuse large B cell lymphoma. Science 2008;319:1676–79. doi: 10.1126/science.1153629 18323416 10.1126/science.1153629

[32] Meininger I, Griesbach RA, Hu D, Gehring T, Seeholzer T, Bertossi A, et al Alternative splicing of MALT1 controls signalling and activation of CD4(+) T cells. Nat Commun. 2016;7:11292 doi: 10.1038/ncomms11292 27068814 10.1038/ncomms11292 PMC4832065

[33] Uren AG, O'Rourke k, Aravind LA, Pisabarro MT, Seshagiri S, koonin EV, et al Identification of paracaspases and metacaspases: two ancient families of caspase-like proteins, one of which plays a key role in MALT lymphoma. Mol Cell 2000;6:961–7. 11090634 10.1016/s1097-2765(00)00094-0

[34] Gewies A, Gorka O, Bergmann H, Pechloff k, Petermann F, Jeltsch kM, et al Uncoupling malt1 threshold function from paracaspase activity results in destructive autoimmune inflammation. Cell Rep. 2014;9:1292–305 doi: 10.1016/j.celrep.2014.10.044 25456129 10.1016/j.celrep.2014.10.044

[35] Bornancin F, Renner F, Touil R, Sic H, kolb Y, Touil-Allaoui I, et al Deficiency of MALT1 paracaspase activity results in unbalanced regulatory and effector T and B cell responses leading to multiorgan inflammation. J Immunol. 2015;194:3723–34. doi: 10.4049/jimmunol.1402254 25762782 10.4049/jimmunol.1402254

[36] Yu JW, Hoffman S, Beal AM, Dykon A, Ringenberg MA, Hughes AC, et al MALT1 protease activity is required for innate and adaptive immune responses. PLoSONE 2015;10:e0127083. 10.1371/journal.pone.0127083 PMC4428694 25965667

