# Supplementary figures and images for: Autocleavage of the paracaspase MALT1 at Arg-781 attenuates NF-κB signaling and regulates the growth of activated B-cell like diffuse large B-cell lymphoma cells
Source: PLoS One. 2018 Jun 28;13(6):e0199779. doi: 10.1371/journal.pone.0199779 (PMC6023146; doi:10.1371/journal.pone.0199779)

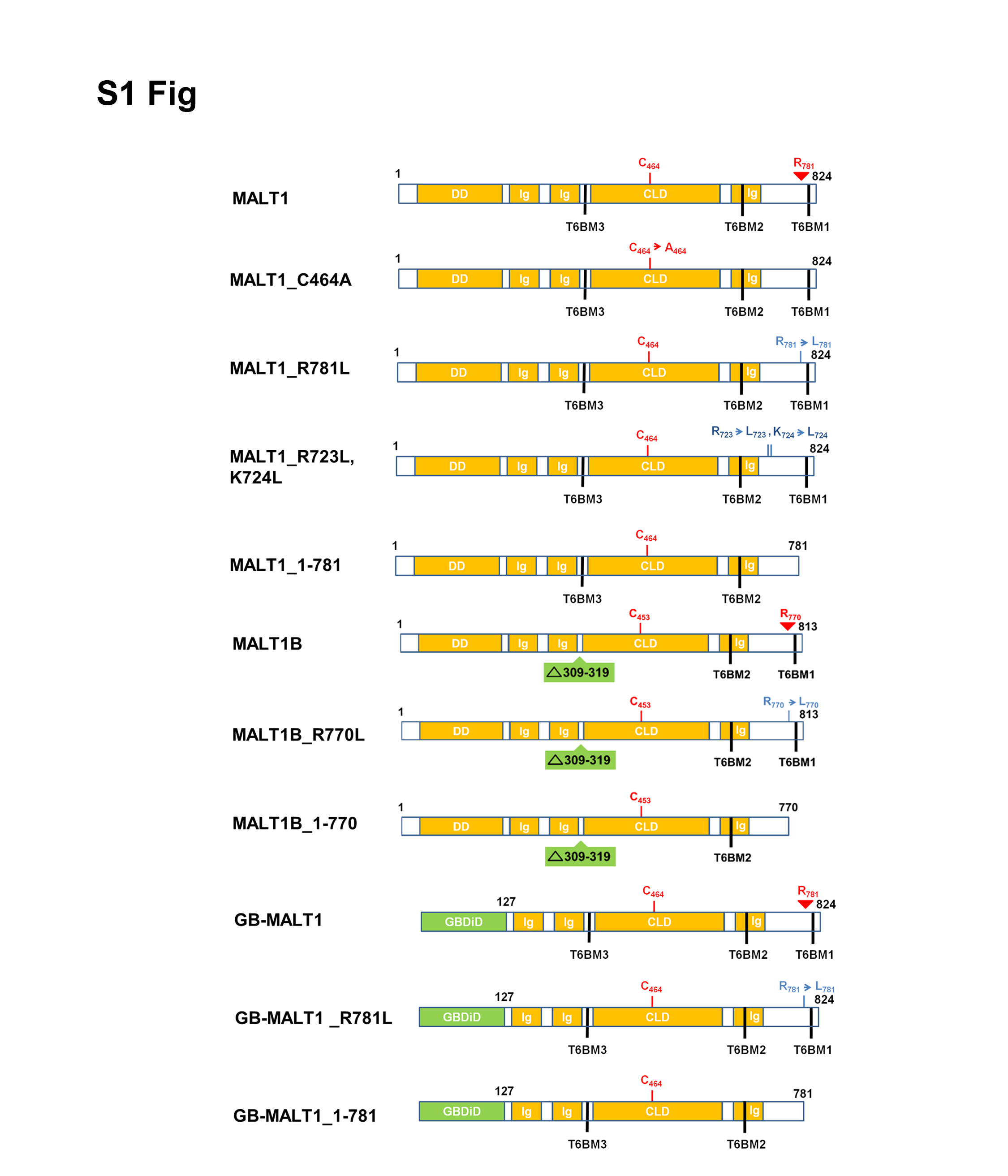

Supplement: S1 Fig — Different domains of MALT1 are shown. DD; Death Domain, Ig: Immunoglobulin-like domain, CLD: Caspase Like Domain, T6BM: TRAF 6 Binding Motif, GBDiD: Gyrase B Dimerization Domain. The catalytic cysteine residue and the autocleavage site are also shown in red. (TIF) [file pone.0199779.s001.tif]

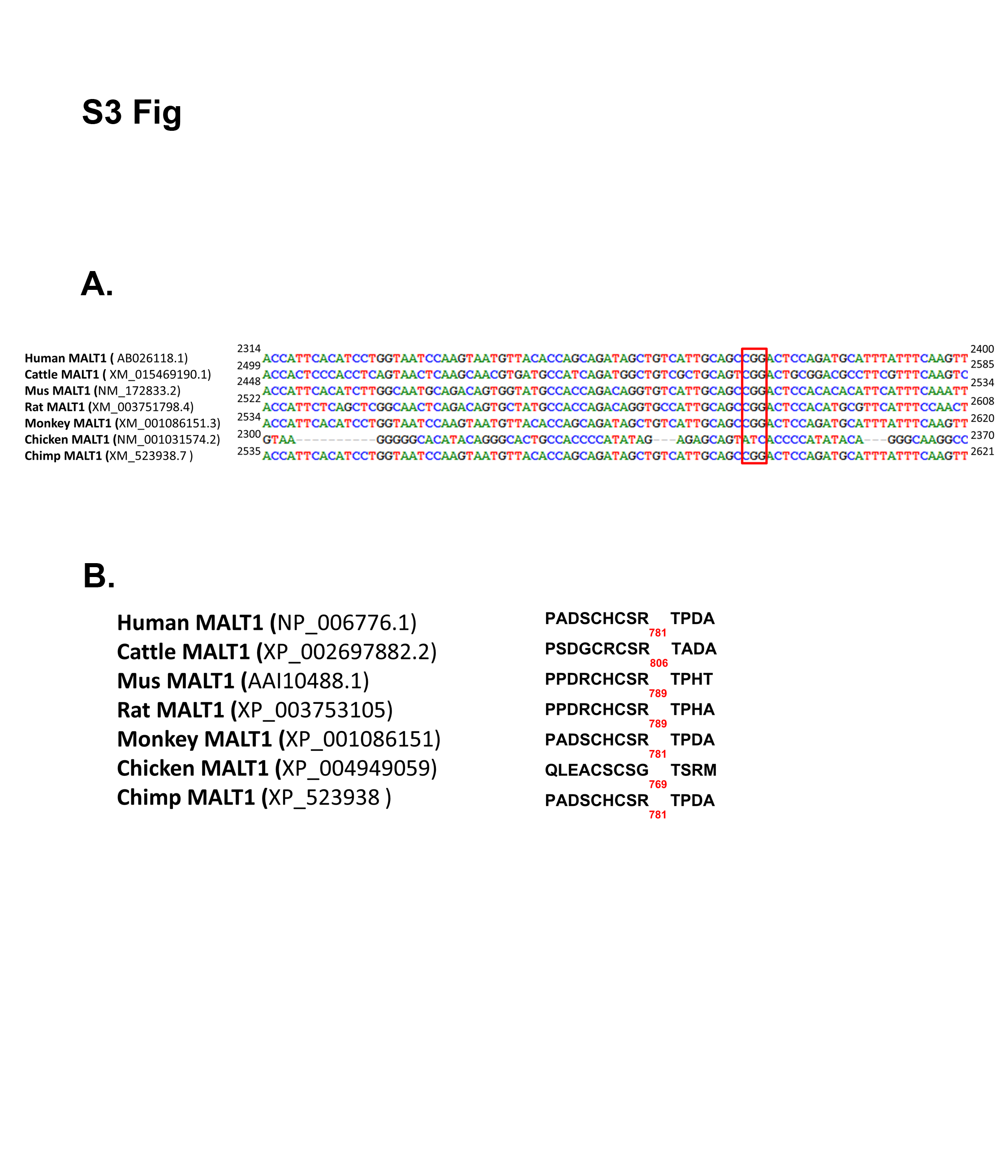

Supplement: S3 Fig — (A) Nucleotide sequence and (B) protein sequence alignment surrounding R781 of MALT1 in different species. (TIF) [file pone.0199779.s003.tif]
