# Supplementary material for: Autocleavage of the paracaspase MALT1 at Arg-781 attenuates NF-κB signaling and regulates the growth of activated B-cell like diffuse large B-cell lymphoma cells
Source: PLoS One. 2018 Jun 28;13(6):e0199779. doi: 10.1371/journal.pone.0199779 (PMC6023146; doi:10.1371/journal.pone.0199779)
Supplement: S2 Fig — BCL10 oligomerizes and exhibits a discrete cytoplasmic filaments when overexpressed. BCL10_L41R with mutation in caspase recruitment domain exhibits a diffused pattern and fails to form filaments. Formation of discrete cytoplasmic filaments was utilized as oligomerization indicator. Phosphorylation of BCL10 correlates with filament formation. (DOCX) [file pone.0199779.s002.docx]

**
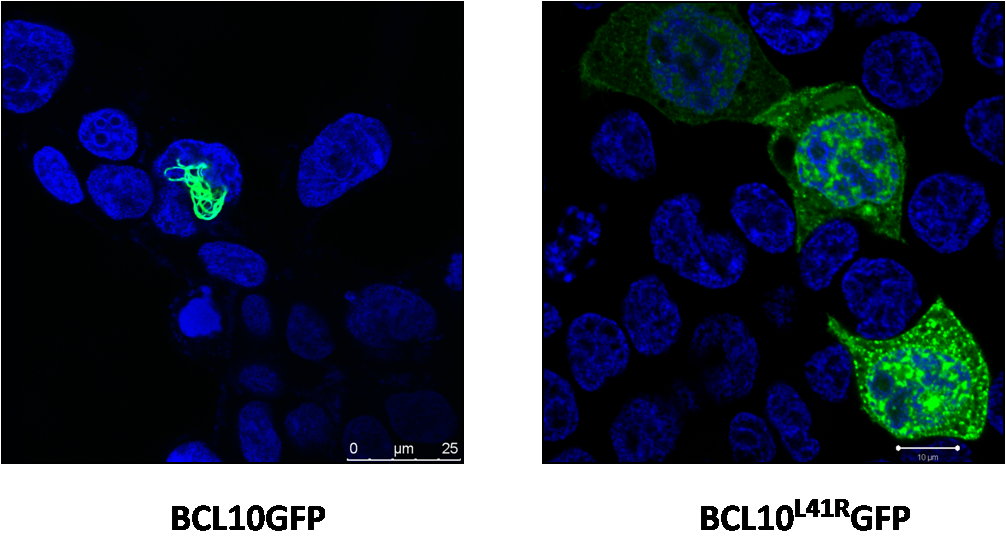
S2 Fig**

**S2 Fig. Fluorescence micrographs of BCL10GFP and BCL10^L41R^GFP**

BCL10 oligomerizes and exhibits a discrete cytoplasmic filaments when overexpressed. BCL10_L41R with mutation in caspase recruitment domain exhibits a diffused pattern and fails to form filaments. Formation of discrete cytoplasmic filaments was utilized as oligomerization indicator. Phosphorylation of BCL10 correlates with filament formation.
