# Supplementary material for: Autocleavage of the paracaspase MALT1 at Arg-781 attenuates NF-κB signaling and regulates the growth of activated B-cell like diffuse large B-cell lymphoma cells
Source: PLoS One. 2018 Jun 28;13(6):e0199779. doi: 10.1371/journal.pone.0199779 (PMC6023146; doi:10.1371/journal.pone.0199779)
Supplement: S1 Text — (DOCX) [file pone.0199779.s004.docx]

**Supplemental Experimental Procedures**

**MALT1 constructs**

pCMV6/XL5/MALT1_C464A, pCMV6/XL5/MALT1 306-824 were described in previous study (26). For **pRc/CMV MALT1**, A 3.7 kb DNA fragment was isolated from *Not I-*cut-Klenow treated pCMV6/XL5/MALT1, and ligated with 5.5 kb *Hind III* -cut-Klnow-treated pRc/CMV. **pCMV6/XL5/MALT1 306-824_R781L, pCMV6/XL5/MALT1 306-824_R723L, K724L, and pCMV6/XL5/MALT1 306-824_1-781**were generated by utilization of a PCR-based site directed mutagenesis method. The primers utilized were as the followings:

5'-TAGCTGTCATTGCAGCCTGACTCCAGATGCATTTATTTC-3' for **pCMV6/XL5/ MALT1 306-824_R781L**; 5'-AAATTAGACATGCATCGAGGTTTGGGACTACTAACTTGCTTTCAAACTTGTCTTATGTCTAATG-3' for **pCMV6/XL5/MALT1 306-824_R723L, K724L** ; 5'-GCAGATAGCTGTCATTGCAGCTAGACTCCAGATGCATTTATTTC-3' for **pCMV6/XL5/MALT1 306-824_1-781**. All mutations were confirmed by DNA sequencing. A 1.0 kb DNA fragment was isolated from *Hind III* and *Ale I*-cut pCMV6/XL5/MALT1 306-824_R781L, pCMV6/XL5/MALT1 306-824_R723L, K724L, and pCMV6/XL5/MALT1 306-824_1-781 individually and ligated with 8.2 kb *Not I*-cut-Klenow-*Hind III*-cut-treated pRc/CMVMALT1, generating **pRc/CMV MALT1_R781L**, **pRc/CMV MALT1_ R723L,K724L** ,and **pRc/CMV MALT1_1-781**.

Two pairs of primers : 5'-AATATTCTGTTTGGATATGCC-3' and 5'-TCACTTGTCATCGTCGTCCTTGTAGTCTTTTTCAGAAATTCTGAGCCT-3' were utilized to amplify a DNA fragment covering aa485-aa824 of MALT1 gene. The DNA fragment was cloned into pGEM-T Easy vector and subjected to sequence analysis. A 1.2 kb fragment was isolated from the *Hind III* and *Not I*-digested mixture of the right clone and ligated with *Hind III* and *Not I*-cut pRc/CMV MALT1, generating **pRc/CMV MALT1-FLAG**.

siRNA-resistant MALT1 clones, including **pCMV6/XL5/MALT1 306-824_R**, **pCMV6/XL5/MALT1 306-824_R781L_R**, and **pCMV6/XL5/MALT1 306-824_1-781_R,** were generated by utilization of a PCR-based site directed mutagenesis method. The primers utilized were 5'-GAACAGAATATTCTGCTGAATCGCTGGTGCTGCGGAATCTACA-3'.

A 1.0 kb DNA fragments was isolated from *Hind III* and *Ale I* cut pCMV6/XL5/MALT1 306-824_R, pCMV6/XL5/MALT1 306-824_R781L_R, and pCMV6/XL5/MALT1 306-824_1-781**_**R separately and ligated with 8.2 kb *Not I*-cut-Klenow-*Hind III*-cut-treated pRc/CMVMALT1, generating **pRc/CMV MALT1_R**, **pRc/CMV MALT1_R781L_R**, or **pRc/CMV MALT1_1-781_R**.

pcDNA3.1**^+^**/C-(K)DYK-MALT1B was digested with *Kpn I*, treated with T4 DNA polymerase to generate blunt ends and digested with *Hind III*. A 1.5 kb DNA fragment was isolated and ligated with a 6.7 kb DNA fragment isolated from *Sgr A1*/Klenow/*Hind III*-treated pRc/CMV MALT1_R, pRc/CMV MALT1_R781L_R, or pRc/CMV MALT1_1-781_R, generating **pRc/CMV MALT1B_R**, **pRc/CMV MALT1B_R770L_R**, or **pRc/CMV MALT1B_1-770_R**. A 2.8 kb DNA fragment was isolated from *Sma I* and *Apa I* digested pRc/CMV MALT1_R, pRc/CMV MALT1_R781L_R, or pRc/CMV MALT1_1-781_R, blunt-ended with Klenow DNA polymerase, and ligated with the 9.3 kb DNA fragment isolated from *Eco RV*-cut pLAS3.Pneo, generating **pLAS3.Pneo MALT1_R, pLAS3.Pneo MALT1_1-781_R, and pLAS3.Pneo MALT1_R781L_R.**

A 1.0kb DNA fragment was isolated from *Hind III* and *Ale I* digested pCMV6/XL5/MALT1 306-824_R and ligated with *Hind III* and *Ale I*-cut pCMV6/XL5/MALT1_C464A, generating pCMV6/XL5/MALT1_C464A_R. A 2.8 kb fragment was isolated from *Sma I* and *Ale I* digested pCMV6/XL5/MALT1_C464A_R and ligated with the 9.3 kb DNA fragment isolated from *Eco RV-*cut pLAS3.Pneo, generating **pLAS3.Pneo MALT1_C464A_R.**

Two pairs of primers: 5'-CCCGGGGCCATGGGCGGCATCAAGGCGTTCGTT-3' and 5'-TGGGTTTACAGTAATCTTGAGCGCACCTTTACGGCGGGT-3' were utilized to amplify a gyrase B DNA fragment. Primers 5’- ACCCGCCGTAAAGGTGCGCTCAAGATTACTGTAAACCCA-3’ and 5’-GAAGATCCTTTGATACCA-3’were utilized to amplify a DNA fragment covering aa128-aa824 of MALT1 gene. Primers 5'-CCCGGGGCCATGGGCGGCATCAAGGCGTTCGTT-3' and 5’-GAAGATCCTTTGATACCA-3’were utilized to amplify the fusion junction of Gyrase B-MALT1 gene. The DNA fragments were cloned into pGEM-T Easy vector and subjected to sequence analysis. A 1.8 kb fragment was isolated from the *Sma I* and *Xba I-cut* mixture of the right clone and ligated with *Sma I* and *Xba I*-cut pCMV6/XL5/MALT1, generating **pCMV6/XL5/GB-MALT1**. A 1.0 kb DNA fragments was isolated from *Hind III* and *Ale I* cut pCMV6/XL5/MALT1 306-824_R781L, and pCMV6/XL5/MALT1 306-824_1-781 separately and ligated with *Hind III* and *Ale I*-cut pCMV6/XL5/GB-MALT1, generating **pCMV6/XL5/GB-MALT1_R781L** and **pCMV6/XL5 GB-MALT1_1-781**.

A 2.8 kb DNA fragment was isolated from *Sma I* and *Apa I* digested pRc/CMV MALT1_R, pRc/CMV MALT1_1-781_R, pRc/CMV MALT1_R781L_R , pRc/CMV MALT1B_R, pRc/CMV MALT1B_1-770_R, or pRc/CMV MALT1B_R770L_R blunt-ended with Klenow DNA polymerase, and ligated with the 8.7 kb DNA fragment isolated from *Eco RV*-cut pAS4.1w.Ppuro-aOn, generating **pAS4.1w.Ppuro-aOn MALT1_R, pAS4.1w.Ppuro-aOn MALT1_1-781_R, pAS4.1w.Ppuro-aOn MALT1_R781L_R, pAS4.1w.Ppuro-aOn MALT1B_R, pAS4.1w.Ppuro-aOn MALT1B_1-770_R, and pAS4.1w.Ppuro-aOn MALT1B_R770L_R.** A 2.8 kb fragment was isolated from *Sma I* and *Ale I* digested pCMV6/XL5/MALT1_C464A_R and ligated with the 8.7 kb DNA fragment isolated from *Eco RV-*cut pAS4.1w.Ppuro-aOn, generating **pAS4.1.Ppuro-aOn MALT1_C464A_R.**
